# A patient with intraoperative awareness history requiring high propofol effect-site concentrations for general anesthesia

**DOI:** 10.1186/s40981-019-0290-6

**Published:** 2019-11-04

**Authors:** Shinju Obara, Yoshie Noji, Takayuki Hasegawa, Chie Hanayama, Rieko Oishi, Masahiro Murakawa

**Affiliations:** 1 Surgical Operation Department, Fukushima Medical University Hospital, 1 Hikarigaoka, Fukushima, Fukushima 960-1295 Japan; 2 Department of Anesthesiology, Fukushima Medical University Hospital, 1 Hikarigaoka, Fukushima, Fukushima 960-1295 Japan; 3 Department of Anesthesiology, Fukushima Medical University School of Medicine, 1 Hikarigaoka, Fukushima, Fukushima 960-1295 Japan

To the Editor,

Intraoperative awareness (IA) occurs in up to 0.12% of patients under general anesthesia [1]. Herein, we describe the case of patient with IA history requiring high propofol effect-site concentrations (PCe) for anesthesia. We obtained a written informed consent from the patient for publication. The patient was a 34-year-old woman (158 cm, 46 kg) who underwent a semi-emergency transcutaneous drainage of an intrapelvic tumor under computed tomography (CT) guidance. She was being administered celecoxib, loxoprofen, and pregabalin for chronic pain. She had undergone multiple operations for neurilemmomas, had experienced IA with unclear details, and desired a complete loss of consciousness for her upcoming operation. In the CT room, we induced anesthesia with fentanyl (150 μg) and propofol via target-controlled infusion (TCI, Diprifusor model, TE-371, Terumo, Tokyo, Japan). We initiated the target plasma concentration of propofol at 4 μg/mL and increased it gradually (Fig. 1). When the patient’s responses to voice, mild prodding, and shaking were lost, the PCe was 6.4 μg/mL and the patient state index (PSi) derived with SedLine® (Masimo, USA) was 79 (PSi 25–50 is recommended during anesthesia). At this stage, fast waves in electroencephalogram were still observed but were not predominant. Spectral edge frequency (SEF), which can be used as a surrogate measure to observe the change in the amount of fast waves, was 12.9 Hz. Following the tracheal intubation using rocuronium (40 mg), we moved the patient to a prone position and installed a mechanical ventilator. We titrated target propofol concentrations in accordance with PSi values and with the patient’s vital signs. The drainage was uneventfully completed. The maximum PCe was 8.0 μg/mL and the total fentanyl dose was 400 μg. Ten minutes after PCe reached 8.0 μg/mL, PSi and SEF were 22 and 10.8 Hz, respectively. The anesthesia duration was 100 min. The patient regained responsiveness to voice when the PCe decreased to 4.0 μg/mL. We did not observe any postoperative complications and the patient was satisfied with the clinical course without any IA. Although no consensus exists on IA, using inhalation anesthetics may produce less IA [2, 3]. However, the CT room was not equipped with the excess anesthesia gas extraction system and this prevented us from using inhaled anesthetics with an anesthesia machine, which is why we chose total intravenous anesthesia. Our patient required much higher PCes than usual (TCIs are 3.0–6.0 μg/mL and 2.0–5.0 μg/mL for anesthesia induction and maintenance, respectively; according to the propofol package insert; Diprivan®, Aspen Japan, Tokyo). The patient was apparently awake, although the probability of loss of response to prodding and shaking right before the patient lost responsiveness was predicted at 99.3% with the response surface model [4]. Therefore, we think she was relatively insensitive to propofol due to pharmacokinetic and/or pharmacodynamic variability, and this may have explained her previous IA. Although pregabalin reportedly reduces propofol requirements [5], our patient required a large propofol dose. Our case highlights the need to be aware of potential propofol insensitivities, especially in patients with IA history.

**Fig. 1:**
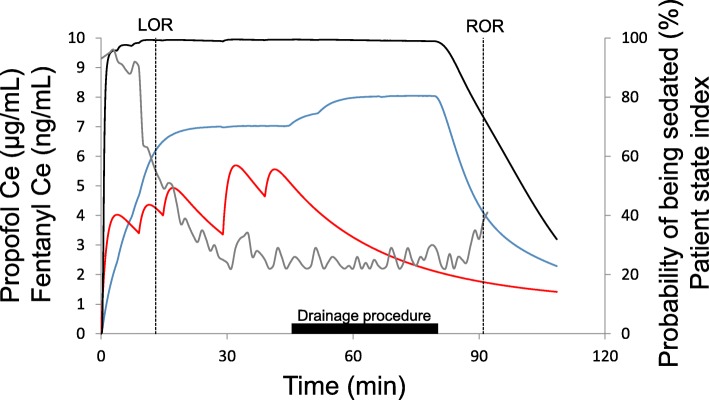
Time courses of propofol effect-site concentration (PCe, blue curve), fentanyl effect-site concentration (FCe, red curve), patient state index (PSi, gray curve), and probability of loss of response to prodding and shaking predicted with a response surface model (black curve) [4]. To use the model, (1) we converted PCes predicted with Diprifusor model to PCes predicted by Schinder model using the infusion record of propofol downloaded from the pump and (2) converted the FCes to remifentanil equivalents using a remifentanil:fentanyl equivalency ratio of 1:1.2. LOR, loss of responsiveness; ROR, return of responsiveness

## Data Availability

Not applicable

## Abbreviations

CT: Computed tomography
FCe: Fentanyl effect-site concentration
IA: Intraoperative awareness
PCe: Propofol effect-site concentration
PSi: Patient state index
TCI: Target-controlled infusion
